# Expression of Non-visual Opsins Opn3 and Opn5 in the Developing Inner Retinal Cells of Birds. Light-Responses in Müller Glial Cells

**DOI:** 10.3389/fncel.2019.00376

**Published:** 2019-08-16

**Authors:** Maximiliano N. Rios, Natalia A. Marchese, Mario E. Guido

**Affiliations:** ^1^Centro de Investigaciones en Química Biológica de Córdoba (CIQUIBIC), CONICET, Facultad de Ciencias Químicas, Universidad Nacional de Córdoba, Córdoba, Argentina; ^2^Departamento de Química Biológica “Ranwel Caputto,” Facultad de Ciencias Químicas, Universidad Nacional de Córdoba, Córdoba, Argentina

**Keywords:** retina, opsin, Müller cells, blue light, retinal ganglion cells, calcium, development

## Abstract

The avian retina is composed of different types of photoreceptors responsible for image and non-image forming tasks: the visual photoreceptor cells (cones and rods), the melanopsin-expressing intrinsically photoresponsive retinal ganglion cells (ipRGCs) and horizontal cells. Furthermore, the non-visual opsins Opn3 (encephalopsin/panaopsin) and Opn5 (neuropsin) have been shown to be expressed in the vertebrate inner retina, responding to blue (BL) and UV light, respectively. Here we investigated the expression and localization of Opn3 and Opn5 in the developing chick retina at different embryonic days (E) as well as in primary cultures of retinal Müller glial cells (MCs). Opn3 and Opn5 mRNAs and proteins appeared as early as E10 although traces of Opn3- and Opn5-like proteins were seen earlier by E7 in the forming RGC layer and in glial cells extending throughout the developing nuclear layer. Later on, at postnatal days 1–10 (PN1–10) a significant expression of Opn3 was observed in inner retinal cells and processes in plexiform layers, together with expression of the glial markers glutamine synthetase (GS) and the glial fibrillary acidic protein (GFAP). Opn3 and Opn5 were found to be expressed in primary MC cultures prepared at E8 and kept for 2 weeks. In addition, significant effects of BL exposure on Opn3 expression and subcellular localization were observed in MCs as BL significantly increased its levels and modified its nuclear location when compared with dark controls, through a mechanism dependent on protein synthesis. More importantly, a subpopulation of MCs responded to brief BL pulses by increasing intracellular Ca^2+^ levels; whereas light-responses were completely abolished with the retinal bleacher hydroxylamine pretreatment. Taken together, our findings show that these two opsins are expressed in inner retinal cells and MCs of the chicken retina at early developmental phases and remain expressed in the mature retina at PN days. In addition, the novel photic responses seen in MCs may suggest another important role for the glia in retinal physiology.

## Introduction

Opsins are photosensitive pigments that bind a retinaldehyde chromophore to form a light-sensitive G protein-coupled receptor able to sense specific wavelength ranges. The vertebrate retina contains a number of different opsins responsible for the photoreception involved in visual functions such as image- and non-image forming tasks (Guido et al., 2010; Meister and Tessier-Lavigne, 2013; Diaz et al., 2016). Classical photoreceptor cells (PRCs) – cones and rods – are responsible for color (diurnal) and black and white (nocturnal) vision, respectively (Meister and Tessier-Lavigne, 2013). PRCs connect through the outer plexiform layer (OPL) to the inner nuclear layer (INL), comprising horizontal cells (HCs), bipolar and amacrine cells, and the INL cells connect with the retinal ganglion cells (RGCs) through the inner plexiform layer (IPL). RGC axons form the optic nerve and send visual information to the brain (Meister and Tessier-Lavigne, 2013). The retina contains diverse types of glial cells, the most abundant being the Müller glial cells (MCs) that extend throughout the inner retina. MCs are responsible for several important physiological activities, both during and after development, significantly contributing to homeostasis and even to more complex processes such as neuronal regeneration (Jorstad et al., 2017; Subirada et al., 2018).

In recent decades, a subpopulation of RGCs expressing the non-visual photopigment melanopsin (Opn4) (Provencio et al., 1998, 2000) was characterized as intrinsically photosensitive RGCs (ipRGCs) in the rodent retina (Hattar et al., 2002). These cells projecting to different brain areas were shown to be involved in setting the biological clock, controlling pupillary light reflexes, suppressing pineal melatonin and other activities (Berson, 2003; Guido et al., 2010; Ksendzovsky et al., 2017; Lazzerini Ospri et al., 2017). It was reported that ipRGCs are also present in chick retina (Contin et al., 2006, 2010; Diaz et al., 2014, 2017) and that they may participate in the regulation of diverse non-visual functions, as observed in blind chicks (Valdez et al., 2012, 2013, 2015). In birds, as well as in other non-mammalian vertebrates, there are two Opn4 genes, the *Xenopus* (Opn4x) and the mammalian (Opn4m) orthologs (Bellingham et al., 2006). Both proteins are expressed in RGCs of chicks very early in development at embryonic day (E) 8 (Verra et al., 2011) while Opn4x is also strongly expressed in HCs by E15 and at later stages (Verra et al., 2011; Morera et al., 2016). Opn4x confers photosensitivity on these HCs (Morera et al., 2016), likely contributing to lateral interaction with PRCs and cooperating with ipRGCs in non-visual activities.

A number of other non-visual opsins/photoisomerases have been reported to be present in the inner retina of mammals such as neuropsin (Opn5) (Tarttelin et al., 2003; Kojima et al., 2011; Nieto et al., 2011) and encephalospin/panaopsin (Opn3) (Halford et al., 2001b). Both opsins are also present in the post-hatching chick retina and brain, specifically in HCs, hypothalamus, and cerebellum (Yamashita et al., 2010; Kato et al., 2016). Furthermore, the putative photoisomerase retinal G protein-coupled receptor (RGR) was shown to be expressed in the inner retina of birds and particularly in Opn4x (+) ipRGCs to modulate retinaldehyde levels in the light, thus maintaining the balance of inner retinal retinoid pools (Diaz et al., 2017).

The goal of the current work was to investigate the onset of expression of the non-visual opsins Opn3 and Opn5 during development in the embryonic chick retina and in primary cultures of MCs. To this end, we first examined the expression of Opn3 and Opn5 at the mRNA and protein levels in whole developing retina at different embryonic (E) stages and in primary MC cultures of E8 retinas. Finally, we evaluated the effect of light exposure on Opn3 expression in retinal cells and the potential photic responses of MCs in culture by Ca^+ 2^ fluorescence imaging.

## Materials and Methods

### Animal Handling

For the different studies performed, chicken embryos (*Gallus gallus domesticus*) (Avico) at different E and at postnatal day 1 (PN1) were used as previously described in Diaz et al. (2016). Eggs were incubated at 37°C with 60% of humid atmosphere (Yonar^®^ Incubators). Chickens were sacrificed by decapitation. All experiments were performed in accordance with the Use of Animals in Ophthalmic and Vision Research of ARVO, approved by the local animal care committee (School of Chemistry, National University of Córdoba; Exp.15-99-39796) and CICUAL (Institutional Committee for the Care and Use of Experimental Animals).

### Retinal Fixation and Sectioning

After enucleation, eyes were hemisected equatorially and the gel vitreous was removed from the posterior eyecup. Samples were fixed for 30 min at 20°C in 4% paraformaldehyde plus 3% sucrose in 0.1 M phosphate buffer, pH 7.4, as described in Diaz et al. (2016). Fixed samples were washed three times in phosphate-buffered saline (PBS; 0.05 M phosphate buffer, 195 mM NaCl, pH 7.4), cryo-protected in PBS plus 30% sucrose, soaked in embedding medium (O.C.T. Compound, Tissue-Tek) for 10 min, and freeze-mounted onto aluminium sectioning blocks. Transverse sections nominally 14 μm thick were cut consistently from the posterior pole of the eye, near the dorsal portion of the pecten, and thaw-mounted on Super-Frost glass slides (Fisher Scientific). Sections were air-dried and stored at −20°C until use.

### Primary Cultures of Müller Glial Cells

Primary cultures of MCs were purified from neural chick E8 retinas dissected in ice-cold Ca^+2^–Mg^+2^-free Tyrode’s buffer containing 25 mM glucose as previously reported (Loiola and Ventura, 2011; Lopez-Colome et al., 2012; Diaz et al., 2014, 2016; Freitas et al., 2016). Briefly, cells were treated with papain (P3125 Sigma–Aldrich) for 25 min at 37°C and deoxyribonuclease I (18047-019 Invitrogen) and rinsed with fetal bovine serum (FBS) 10% and Dulbecco’s modified Eagle’s medium (DMEM). After dissociation, the cells in suspension were seeded in Petri dishes and grown in DMEM supplemented with 10% FBS for at least 1 week; then, cultures were replicated using trypsin in new Petri dishes and allowed to grow for another week in DMEM supplemented with 10% FBS at 37°C under constant 5% CO_2_-air flow in a humid atmosphere.

### Light Treatment in Primary Cultures of Retinal Cells

Primary cultures of MCs were pre-incubated with 9-*cis-*retinal 0.6 μM for 1 h in DMEM. Cultures were then divided into three groups: darkness control (dark), blue light (BL) stimulation (470–490 nm, peak at 480 nm) for 1 h (BL 1 h, LED of 68 μW/cm^2^), and darkness condition for 1 h after the BL stimulation (1 h post-BL). In some experiments, cells were treated with 50 μg/mL cycloheximide (CHD) (Sigma–Aldrich) to inhibit protein synthesis, for 1 h before BL stimulation or at the beginning of the BL stimulus for the 1 h post-BL group. Cells were used for immunocytochemistry and protein purification.

### Immunohistochemistry

Retinal sections were fixed with 4% paraformaldehyde, washed in PBS for 10 min, and permeabilized with Triton 100 0.1% for 15 min according to Diaz et al. (2016) with modifications. Samples were subsequently treated with blocking buffer [PBS supplemented with 3% bovine serum albumin (BSA) 0.1% Tween 20, and 0.1% NaNO_3_] for 40 min and incubated overnight at 4°C with the respective antibodies as indicated in Supplementary Table 1. The retinal sections were rinsed three times in washing buffer (PBS with Tween 20 0.1%) and finally incubated with secondary antibodies (Jackson Dylight^TM^ 488-conjugated AffiniPure Donkey Anti-Mouse or Dylight^TM^ 549-conjugated AffiniPure Donkey Anti-Rabbit at 1:1000 dilution) for 1 h at room temperature (RT). The samples were also incubated with 4′,6-diamidino-2-phenylindole (DAPI) stain (3 μM). Coverslips were finally washed thoroughly and visualized by confocal microscopy (FV1200; Olympus, Tokyo, Japan).

### Western Blot

Homogenates of chick embryonic retinas and primary MCs cultures were resuspended in RIPA buffer [50 mM Tris–HCl, pH 8.0, with 150 mM sodium chloride, 1.0% Igepal (NP-40), 0.5% sodium-deoxycholate, and 0.1% sodium dodecyl sulfate] containing protease inhibitors (Sigma–Aldrich) and processed for western blot (WB) according to Verra et al. (2011) and Nieto et al. (2011). Homogenates were resuspended in sample buffer and separated by SDS-gel electrophoresis on 12% polyacrylamide gels (50 mg total protein/lane), transferred onto Polyvinylidenefluoride (PVDF) membranes, blocked for 1 h at RT with 5% BSA in PBS, and then incubated overnight at 4°C with specific antibodies (Supplementary Table 1) in the incubation buffer (3% BSA, 0.1% Tween 20, 1% glycine, 0.02% sodium azide in PBS). Membranes were washed three times for 15 min each in washing buffer (0.1% Tween 20 in PBS) and incubated with the corresponding secondary antibody in the incubation buffer during 1 h at RT followed by three washes with washing buffer for 15 min each. Membranes were scanned using an Odyssey IR Imager (LI-COR Biosciences). After incubation with the specific antibodies, membranes were stripped with NaOH 1 M for 5 min and incubated in blocking buffer containing α-tubulin antibody.

### Immunocytochemistry

Cultured cells were fixed for 15 min in 4% paraformaldehyde in PBS and washed in PBS, treated with blocking buffer (3% BSA, 0.1% Tween 20, 1% glycine, 0.02% sodium azide in PBS), and incubated overnight with the primary antibodies (Supplementary Table 1). They were then rinsed in PBS and incubated with secondary antibodies from Jackson (1:1000) for 1 h at RT. Samples were incubated with DAPI (3 μM). Coverslips were finally washed thoroughly and visualized by confocal microscopy (FV1200; Olympus, Tokyo, Japan) (Morera et al., 2012).

### RNA Isolation and RT-PCR

Total RNA from whole retina and MC cultures was extracted following the method of Chomczynski and Sacchi using the TRIzol^TM^ kit for RNA isolation (Invitrogen). RNA integrity was checked and quantified by UV spectrophotometry (Epoch Microplate Spectrophotometer, Biotek). Finally, 2 μg of total RNA was treated with DNAse (Promega) to eliminate contaminating genomic DNA. cDNA was synthesized with M-MLV (Promega) using Random Primers (Promega) as previously described in Diaz et al. (2016).

The oligonucleotide sequences used for PCR from the *G. gallus* sequences were as follows:

*Opn3*:

Forward GCCTCTTCGGGATCGTTTCA
Reverse ATGTGATAGCCCGCCAAGAC

*Opn5*:

Forward GACTTAAAGCTGCGGTGCTC
Reverse TCCCAGAGTTGAAAGAATCCCAAT

*TATA-binding protein* (*TBP*):

Forward TGGCACACGAGTAACAAGAG
Reverse CCTTGAGCGTCAGGGAAATAG

### Polymerase Chain Reaction

Initial denaturation step of 1 min at 94°C, 35 cycles of 60 s at 94°C, 50 s at 60–65°C, 90 s at 72°C, and a final 5 min elongation step at 72°C. Amplification products were separated by 2% agarose gel electrophoresis and visualized by ethidium bromide or sybersafe (Invitrogen^TM^) staining.

### Calcium Imaging by Calcium Orange AM Fluorescence Microscopy

Cells were grown in an 8-well Lab-Tek recording chamber (Nunc^TM^, NY, United States) in a colorless DMEM (GIBCO). On the day of the experiment, MCs were incubated with 0.1% of pluronic acid F-127 and the Ca^2+^ indicator dye Calcium Orange AM 5 μM (Invitrogen–Molecular Probes) in a colorless DMEM for 1 h at 37°C, under darkness condition. The fluorescence imaging technique was performed by using the Ca^2+^-sensitive indicator excited at 543 nm with a laser coupled to a confocal microscope (Olympus FluoView-1000). The emitted fluorescence was captured every 2 s, using a PlanApo N 60 × Uplan SApo oil-immersion objective (NA: 1.42; Olympus). The 12 bit 4 × 4 binned fluorescence images for each photo were used to quantify fluorescence levels in the cells using ImageJ; the mean fluorescence intensity in each cell was background-corrected by subtracting the mean fluorescence of an area with no cells (only cells showing positive fluorescence pre-stimuli were considered for the analysis). The mean intensity of individual cells was measured in each captured image series. Changes in fluorescence levels were quantified as the ratio between each relative intensity level measured after a BL stimulus of 68 μW/cm^2^ for 20 s (*F*) and the mean of intensities of serial pictures before stimulation (*F*_*o*_), the same measurements were quantified in the background to correct *F*/*F*_*o*_ for each cell, as described in Morera et al. (2016). Fluorescence intensities during stimulation were not considered for the analyses and are shown as arbitrary values of *F*/*F*_*o*_ = 1. In some experiments, MC cultures were treated with 30 mM hydroxylamine (Taurus) for 1 h in DMEM to block the retinal binding to opsins or treated with 2 μM Ionomycin (Sigma–Aldrich) as positive control. HEK-293 cells, that do not express non-visual opsins, were used as negative controls and stimulated with a BL pulse or Ionomycin. Values of *F*/*F*_*o*_ are not linearly related to changes in [Ca^+2^]*_*i*_* but are intended to provide a qualitative indication of variations in [Ca^+2^]*_*i*_*. No significant vehicle effects or changes in focus were detected. Responses were considered significant when the ratio at the peak differed from the baseline levels by at least 20%.

### MTT Assay

Mcs enriched cultures were replicated in 96-well plates and grown for 4 days at 37°C. Cells were divided into two groups: dark and BL for 1 h. Cells in darkness were used as basal controls. After treatment, the medium was removed and replaced by 10% FBS in DMEM and cells were kept in darkness for 24 h at 37°C. Then, 10 μL of MTT reagent (5 mg/mL; Sigma) was added to each well, plates were further incubated for 2 h at 37°C as described by Wagner et al. (2018) followed by addition of 100 μL of DMSO:isopropanol (1:1, v/v) and incubation for a few minutes at RT, protected from light. Samples were analyzed at a wavelength of 570 nm with a reference at 650 nm in an Epoch Microplate Spectrophotometer.

### Statistics

Statistical analyses involved a *t*-test or one-way analysis of variance (ANOVA) followed by Bonferroni *post hoc* comparison when appropriate. Otherwise, when normal distribution or homogeneity of residuals was infringed, Mann–Whitney (M–W) or Kruskal–Wallis (K–W) tests were used with pairwise comparisons performed by the Dunn’s test when appropriate. In all cases significance was considered at *p* < 0.05.

## Results

To investigate expression of Opn3 and Opn5 in the developing retina of chickens, we evaluated their expression at different embryonic stages in the whole retina and in primary cultures of MCs at early embryonic days. BL-driven responses in MCs were assessed by WB, immunochemistry, and Ca^+ 2^ fluorescence imaging.

### Expression of Opn3 and Opn5 in the Developing Retina

**FIGURE 1:**
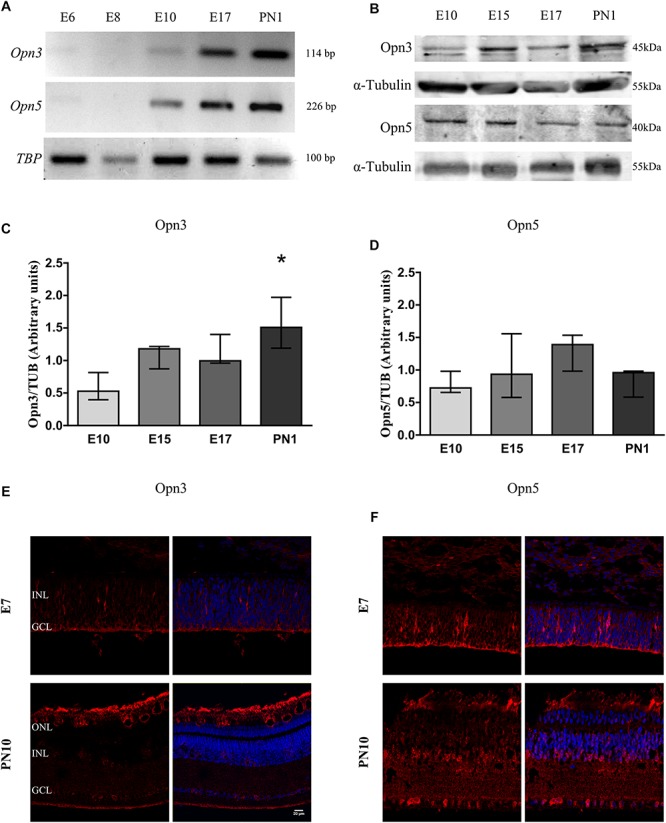
Temporal profiles of Opn3 and Opn5 expression in the developing chicken retina. **(A)** Analysis of *Opn3* and *Opn5* mRNA expression (with *TBP* as housekeeping gene) in the whole chicken retina from embryonic day 6 (E6) to post-natal day 1 (PN1). **(B–D)** Analysis of protein expression of Opn3 and Opn5 in the whole chicken retina from E10 to PN1 by WB. **(B)** Immunoblots show two bands of ∼43–45 kDa for Opn3 protein, whereas a single band was observed at ∼40 kDa for Opn5 protein. **(C)** The histogram shows the median with range for Opn3 levels in the whole chicken retina from E10 to PN1. The K–W test revealed a significant effect of developmental stage [*H*_(__3__)_ = 8.13, ^∗^*p* < 0.05], ^∗^Values at PN1 differ from those at E10 stage (*n* = 3/group). **(D)** The graph shows the median with range for Opn5 levels in the whole chicken retina from E10 to PN1. The K–W test revealed no significant effect of development [*H*_(__3__)_ = 3.97, *p* > 0.05 (*n* = 3/group)]. **(E,F)** Immunohistochemical labeling of Opn3- and Opn5-like proteins, respectively, in retinal sections at E7 and PN10. Opn3- and Opn5-like proteins are stained in red and DAPI in blue. Retinal sections were visualized by confocal microscopy at 60× magnification. Scale bar = 20 μm.

**FIGURE 2:**
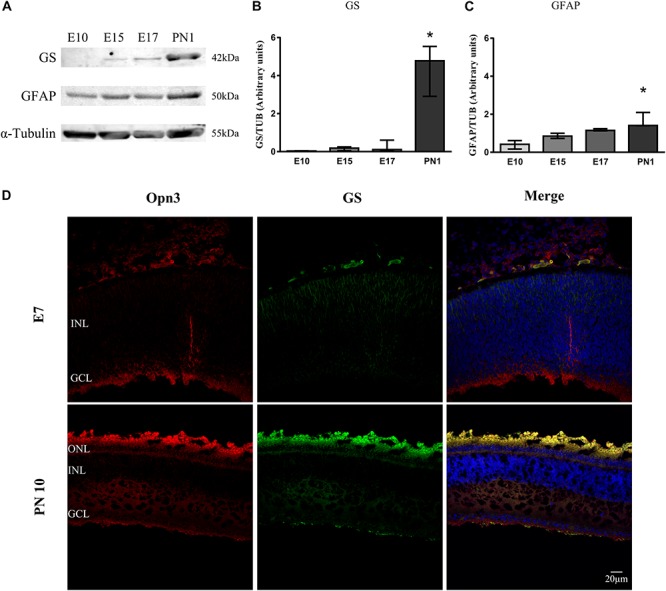
Temporal profile of Müller glial cells markers in the developing chicken retina. **(A)** Analysis of glutamine synthetase (GS) and glial fibrillary acidic protein (GFAP) levels of whole chicken retinas from embryonic day 10 (E10) to post-natal day 1 (PN1) assessed by WB; immunoblots show bands at ∼42 and 50 kDa, respectively. **(B,C)** The histograms show the median with range of GS and GFAP levels in the whole chicken retina from E10 to PN1. The K–W test revealed a significant effect of development on glial markers’ protein expression [*H*_(__3__)_ = 9.36, *p* < 0.001 for GS and *H*_(__3__)_ = 10.38, *p* < 0.0001 for GFAP]. PN1 values differ from those at E10 stage (^∗^*p* < 0.05, *n* = 3/group). **(D)** Immunohistochemistry showing co-localization of Opn3- (red) and GS-like proteins (green) in the chicken retina at E7 and PN10. At E7 Opn3-like protein is localized mainly in the ganglion cell layer (GCL) with low GS expression. In the PN10 stage, Opn3-like protein shows a more ubiquitous localization in the inner nuclear layer (INL) and an increase in the expression with GS at the GCL. Retina sections were visualized by confocal microscopy at 60× magnification. Scale bar = 20 μm.

Figures 1, 2 show that Opn3 and Opn5 mRNAs (Figure 1A) and proteins (Figures 1B–F, 2D) from samples of the whole developing retina collected at different embryonic days appeared as early as E10. However, traces of Opn3- and Opn5-like proteins were also seen earlier by E7 mainly in the first-appearing cells in the RGC layer and extending throughout the developing nuclear layer (Figures 1E,F, 2D). A significant effect of developmental time for Opn3 expression along the embryonic stages up to PN1 was found (*p* < 0.05 by K–W test) (Figure 1C) whereas *post hoc* comparisons indicated that levels of Opn3 protein at PN1 differed from those at E10. By contrast, no significant differences were observed in Opn5 expression across time examined from E10 to PN1 (Figure 1D). Controls of antibody specificity against Opn3 and Opn5 proteins are shown in Supplementary Figures 1, 2, respectively.

In later stages (PN10), positive immunoreactivity for Opn3-like protein was observed in inner retinal cells and plexiform layers in close proximity to the glial marker glutamine synthetase (GS) (Figure 2D). As shown in Figure 2, the glial fibrillary acidic protein (GFAP) marker was detectable by WB in samples of the whole retina as early as E10 whereas GS was visualized at a later stage (E15 and later on); nevertheless, GS-like protein was observed as early as E7 by IHC (Figure 2D), when Opn3 and Opn5 were also visualized.

A significant temporal effect on GS expression along development was visualized (*p* < 0.05 by K–W test) with the highest GS levels at PN1 (*p* < 0.01) compared with those at E10 (Figure 2B). Also, there was a significant temporal effect for GFAP expression along the embryonic days (*p* < 0.05 by K–W test). Pairwise comparisons revealed that GFAP levels at PN1 (*p* < 0.0001) differed from those at E10 (Figure 2C).

### Expression of Opn3 and Opn5 in Müller Glial Cell Primary Cultures. Light Effects

**FIGURE 3:**
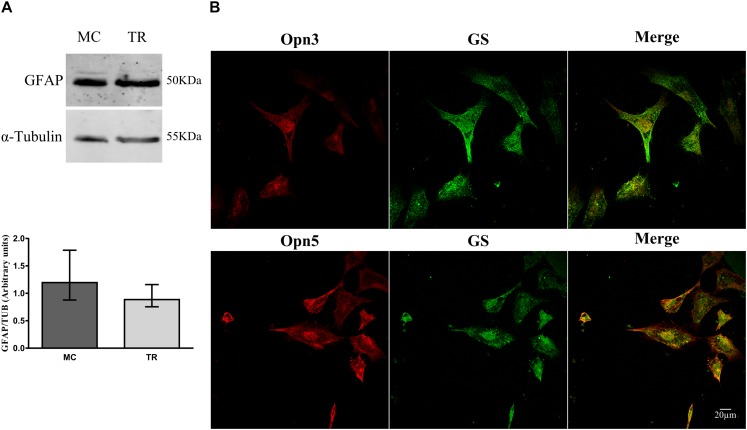
Characterization of primary cultures of Müller glial cells. **(A)** Analysis of GFAP levels by WB in primary cultures of Müller glial cells and of total retina (TR) prepared at E8, immunoblots show bands at ∼50 kDa. The histograms show the median with range for GFAP levels in primary cultures of MC and TR (*n* = 4/group). Primary cultures were highly enriched in Müller glial cells as defined by morphological cell identification (non-neuron-like morphology) and glial marker expression. **(B)** Primary cultures of Müller cells were labeled for Opn3- or Opn5- (red) and GS-like proteins (green) and visualized by confocal microscopy. Müller cells showed ubiquitous and generalized localization of Opn3 and Opn5. Scale bar = 20 μm.

**FIGURE 4:**
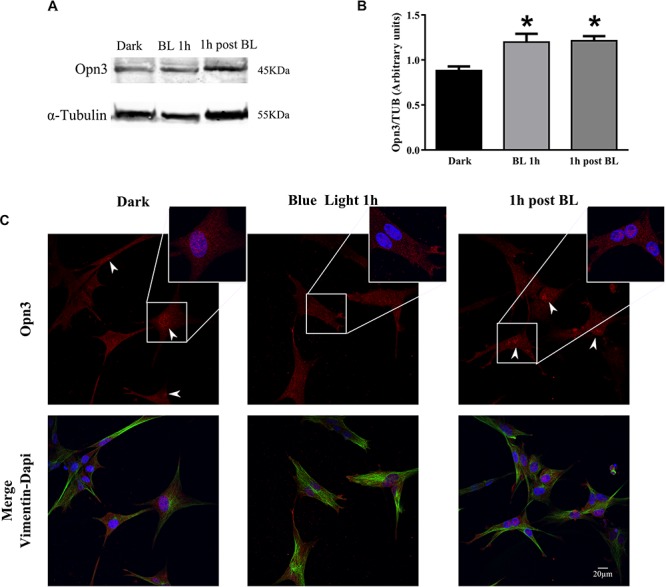
Effect of blue light on Opn3 protein in primary cultures of Müller glial cells. **(A)** Analysis of Opn3 protein in Müller cells at different light conditions by WB. Immunoblots show two bands of ∼43–45 kDa for Opn3 protein in the three experimental conditions: dark, blue light for 1 h (BL 1 h), and 1 h post-BL. **(B)** The histograms show the media ± SEM of Opn3 levels in the three illumination conditions. Increased Opn3 protein levels in Müller glial cells cultures were observed for 1 h of BL stimulation and 1 h after BL exposure over the dark control [^∗^*p* < 0.01 by one-way ANOVA *F*(_2,9_) = 8.48, *n* = 3–5/group]. **(C)** Localization of Opn3 immunoreactivity in Müller cell cultures by ICC for Opn3 (red), the intermedium filament marker Vimentin (green), and DAPI (blue), further magnified in the insets. Opn3-like protein was localized in inner cellular membranes with a strong labeling surrounding the nucleus in darkness and 1 h post-BL conditions, where nuclear accumulations were detected (see insets for higher magnification). By contrast, cell cultures exposed to BL for 1 h did not show substantial nuclear-like accumulation of Opn3 immunofluorescence. Scale bar = 20 μm.

We next investigated expression of Opn3 and Opn5 early in development, in primary cultures highly enriched in MCs prepared at E8 and kept for 2 weeks. Glial cell cultures exhibited the typical glial morphology and expressed higher levels of the characteristic glial marker GFAP by WB [median value, in arbitrary units, 1.20 vs. 0.89 in MCs and total retina (TR) cultures, respectively, Figure 3A], and showed positive immunoreactivity for the glial markers GS and Vimentin by ICC (Figures 3B, 4C). Double immunolabeling for Opn3- and Opn5-like proteins together with the glial marker GS was observed in MCs cultures (Figure 3B). It is noteworthy that both opsins exhibited a particular subcellular pattern of labeling in GS (+) MCs (merge). Immunoreactivity for the two photopigments was seen in the whole cell with some nuclear staining; similar subcellular distribution has been reported in different cell lines as described in The Human Protein Atlas^1^.

In another series of experiments, MCs cultures were exposed to BL of 68 μW/cm^2^ for 1 h (BL), other cultures were later returned to the dark for 1 h (1 h post-BL), and controls maintained in the dark for different pharmacological treatments (Figure 4 and Supplementary Figure 3). As shown in Figure 4, BL exposure significantly increased the levels of Opn3 protein in the MCs cultures (Figures 4A,B) as seen in 1 h BL or 1 h post-BL cultures compared with dark controls. The one-way ANOVA revealed a significant effect of light condition on Opn3 levels (dark, BL 1 h, and 1 h post-BL) (*p* < 0.01). *Post hoc* comparisons indicated that Opn3 levels for 1 h BL and 1 h post-BL conditions differed from controls maintained in the dark (*p* < 0.05).

In addition, BL exposure caused a notable change in the intracellular localization of this opsin as shown in Figure 4C. As denoted with white arrowheads and further magnified in the insets (Figure 4C), immunoreactivity associated to Opn3 was distributed all through the cellular body and nuclei of both dark conditions (dark control and 1 h dark post-BL) whereas for BL-treated cultures, a diminished nuclear localization and a higher immunoreactivity in processes resembling proto-end feet were visualized.

In further experiments carried out in the presence of the protein synthesis inhibitor CHD shown in Supplementary Figure 3, it was demonstrated that if protein synthesis is blocked, no light induction is observed in 1 h BL- or 1 h post-BL-treated cultures with similar protein levels to those found in the dark (Supplementary Figures 3A,B). Results strongly suggest that such mechanism depends on the *de novo* synthesis of protein. The K–W test revealed that there was no significant effect on Opn3 expression under the different light conditions examined with CHD pretreatment. In addition, Supplementary Figure 3C shows that Opn3 immunolabeling remained concentrated in nuclear compartments and all through the cell body in cultures treated with CHD, even after BL exposure (Supplementary Figure 3C) and no re-localization of Opn3-like immunofluorescence was observed in any illumination condition (dark, BL, or 1 h post-BL).

In another series of control experiments, and to discard any deleterious effect of bright BL exposure in retinal cell cultures, cell viability by MTT in dark or photic-exposed cells to BL for 1 h was assessed 24 h after the stimulus (Supplementary Figure 4). No significant differences were found in cell viability according to MTT absorbance of MCs cultures between dark and 1 h BL conditions.

### Light Responses in Primary Müller Glial Cell Cultures

**FIGURE 5:**
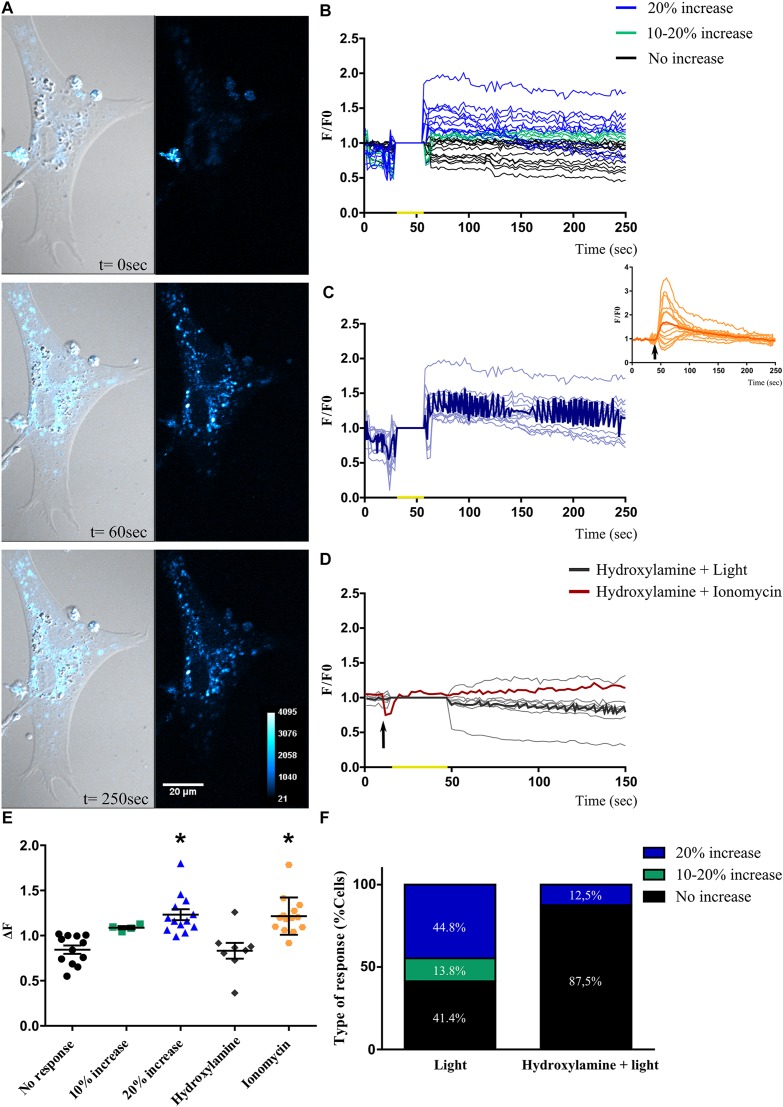
Blue light-induced changes in intracellular Ca^2+^ levels in Müller glial cells. **(A)** Representative Müller glial cells kept in culture for 2 weeks loaded with Calcium Orange AM and displaying a significant increase in intracellular Ca^2+^ fluorescence levels after a blue light pulse (BL) of 20 s and visualized at 60 and 250 s (left panels). The Ca^2+^ response is shown in a pseudo color scale with the highest response in white (right panels). Scale bar = 20 μm. **(B)** Graphical representation showing the *F*/*F*_*o*_ ratio for different Ca^2+^ responses in Müller glial cells when exposed to a single brief BL pulse of 68 μW/cm^2^ for 20 s (yellow mark). Responses are divided into: (i) cells that did not respond to BL (black lines); (ii) cells that responded with fluorescence increase between 10–20% to BL (green lines); (iii) cells that responded with an increase in fluorescence >20% to BL (blue lines). **(C)** Graphical representation of the *F*/*F*_*o*_ ratio for changes in fluorescence levels in cells that responded with 20% increase in fluorescence to the BL stimulus (yellow mark). The dark blue line represents the average response of photosensitive cells. Inset shows Müller glial cells responses to ionomycin stimulation (2 μM) (arrow). **(D)** Graphical representation indicating the *F*/*F*_*o*_ ratio for the changes in fluorescence levels in cells pre-treated with the retinal bleacher hydroxylamine (30 mM) and stimulated with BL pulse (yellow mark; gray lines) and the ionophore ionomycin (2 μM) (arrow; red line). **(E)** Graphical representation of relative fluorescent Ca^2+^ levels (Δ*F*) in Müller glial cells when exposed to a single brief BL pulse: black, cells that did not respond to BL; green, cells that responded with a fluorescence increase between 10 and 20% to BL; blue, cells that responded with an increase in fluorescence >20% to BL; gray, cells pre-treated with the retinal bleacher hydroxylamine (30 mM); and orange, cells stimulated with ionomycin (2 μM). The graph shows individual values with the media ± SEM. A one-way ANOVA revealed a significant effect of treatment of light or ionomycin [*F*(_4,46_) = 10.63, ^∗^*p* < 0.0001) compared with the no-increase in Ca^2+^ levels group (51 cells from eight independent experiments). **(F)** Graphical representation for the percentage of each type of response in Müller glial cells pre-treated or not with hydroxylamine. The Ca^2+^ increase triggered by the brief BL pulse is abolished with the retinal bleacher, suggesting an opsin-mediated response.

For all retinal photoreceptors characterized, a common feature found leading to intrinsic photosensitivity is related to significant changes in intracellular Ca^2+^ levels differentially causing cell hyperpolarization or depolarization, respectively (Qiu et al., 2005; Sekaran et al., 2005; Contin et al., 2010; Nieto et al., 2011; Morera et al., 2016; Diaz et al., 2017). Taking these observations into consideration, and in order to test whether primary MCs cultures expressing non-visual opsins (shown in Figures 3, 4 and Supplementary Figure 3) may respond to BL, we assessed changes in intracellular Ca^2+^ levels by fluorescent imaging with calcium orange in cell cultures after photic stimulation. At first, when MCs cultures were exposed to brief light pulses of white (1000 lux), red or BL of 68 μWatt for 10 s, no responses were evoked (data not shown); however, a BL pulse of 20 s was able to promote significant increases (>20%) in relative intracellular Ca^2+^ levels in individual MCs (Figures 5A,B). Moreover, Figure 5C shows the light-evoked responses in Ca^2+^ fluorescence for the subpopulation displaying ≥20% increase responses, with the dark blue recording representing the average of all evoked responses. In this subset of glial cells, positive light responses to a 20 s BL pulse persisted for several minutes, ranging from 100 to 300 s. In addition, the Ca^+ 2^ ionophore ionomycin (2 μM) was used as a positive control in MCs, promoting an acute and transient peak of Ca^+ 2^ increase as indicated in the inset of Figure 5C. As shown in Figures 5E,F, 41.40% of cells in the culture did not respond to BL stimulation at the intensity applied (Figure 5E, black circles); however, 58.60% of cells left did respond according to the following distribution: 14% of cells did respond, showing a 10–20% increase in Ca^2+^ fluorescence levels over the basal threshold (Figures 5E,F, green squares), while another subpopulation of cells (∼44.80%) responded with an increase in Ca^2+^ levels >20% (Figures 5E,F, blue triangles). It is known that most opsin photopigments identified used a vitamin A-derivative retinaldehyde as chromophore (Diaz et al., 2016). To further investigate the specificity of the BL responses observed in MCs cultures, in other series of control experiments, we proceeded to bleach the retinaldehyde by pretreating cultures with hydroxylamine before BL stimulation. As shown in Figure 5D, light-responses associated to changes in intracellular Ca^2+^ were completely abolished with the retinal bleacher hydroxylamine (30 mM) pretreatment, resulting in 87.5% of non-responding cells. Relative fluorescent Ca^2+^ levels (Δ*F*) analysis indicates significant different responses of the higher than 20% increase subpopulation and ionomycin stimulated group compared with fluorescence values in non-responding retinal MCs (*p* < 0.0001 by ANOVA, Figure 5E).

Although not all glial cells in the cultures responded to light in the experimental conditions tested, other controls were carried out to assess the specificity of the observed photic responses, by assessing Ca^2+^ fluorescence imaging in HEK-293 cells exposed to bright BL (68 μW/cm^2^) (Supplementary Figure 5). No BL responses were found in any cell of the cultures exposed to different intensities and durations (data not shown). HEK-293 cells not expressing Opn3 and Opn5 [our results, Yamashita et al. (2010) and Sugiyama et al. (2014) were unable to respond to BL under the experimental conditions tested (light blue lines; average: dark blue) but did so in the presence of 2 μM ionomycin (orange lines, average: red) (Supplementary Figure 5A)]. This pharmacological treatment, known to promote the intracellular mobilization of Ca^+ 2^, did elicit significant increases in the Ca^2+^ indicator fluorescence (Supplementary Figure 5B).

## Discussion

In addition to the cone and rod photopigments (Opn1 and Opn2) involved in purely visual functions relating to day/night vision and image forming processes, a wide range of non-visual opsins has been characterized in vertebrates over the last decades whose functions are not fully known (Peirson et al., 2009; Guido et al., 2010; Nagata et al., 2010; Terakita and Nagata, 2014; Leung and Montell, 2017; Dalesio et al., 2018); among these, Opn3, Opn4, and Opn5 are the most recently identified. In addition to mammals and other vertebrates (Bertolesi et al., 2014), ipRGCs expressing Opn4x and Opn4m were also shown to be present in chick retina (Contin et al., 2006, 2010; Diaz et al., 2014, 2016), appearing very early in development at E8 (Verra et al., 2011). Opn4x is also strongly expressed in HCs by E15 and at later stages (Verra et al., 2011; Morera et al., 2016), conferring photosensitivity on these HCs through a photocascade involving the Gq protein, activation of phospholipase C, Ca^2+^ mobilization, and GABA release (Morera et al., 2016).

Here, we demonstrate that the non-visual opsins Opn3 and Opn5 are expressed in inner retinal cells of chick retina at early phases of development and remain expressed in the mature retina at PN1 and PN10. Moreover, our results indicate that these opsins are expressed particularly in glial cells, considering the similar expression pattern of the glial marker GS and Opn3 in the developing and mature retina and their expression in enriched MCs cultures.

Opn3 was originally designated as encephalopsin and then as panopsin, since it was found to be expressed within the retina and in extra-retinal tissues such as brain, testis, liver, and lung (Blackshaw and Snyder, 1999; Halford et al., 2001a, b; Tarttelin et al., 2003). In the human retina, Opn3 protein is expressed in the different neural layers including the GCL (Halford et al., 2001a); in the chick, it was more recently shown that two Opn3-related proteins (cOpn3 and cTMT) are present in PN retinal HCs, hypothalamus, and cerebellum (Kato et al., 2016). Here, we found that Opn3 mRNA and protein were detected in the whole chick retina at early stages around E7–10 up to PN1 and PN10 (Figures 1, 2). Opn3 expression was localized at first in cells of the forming GCL and extending throughout the inner developing retina; whereas later on, Opn3 immunoreactivity was also visualized in INL and their plexiform layers (IPL and OPL) of retinal sections (Figures 1, 2).

Remarkably, Opn3 and glial markers show matching expression curves along the embryonic stages. Our present results indicate a gradual increase of the glial structural marker GFAP expression up to the PN1; whereas at this point a substantial increase in GS expression was found which correlates with a functional visual retina (Figure 2; Thanos and Mey, 2001). In fact, GS at PN10 is observed as a ubiquitous immunolabeling at the ONL, IPL, and GCL where glutamate transmission is essential for retinal activity (Meister and Tessier-Lavigne, 2013). In this sense, MCs are fully specified near the last stages of development, along which these cells are becoming mature by establishing their polarity and functional domains throughout the retinal layers (MacDonald et al., 2017). It has been proposed that synaptic activity plays a crucial role in shaping these functional domains, as Ca^+2^ responses in MCs vary from acetylcholine- to glutamate-dependent activities across the developmental stages (Rosa et al., 2015; MacDonald et al., 2017). Interestingly, we observed similar distribution patterns of Opn3 and GS in the chicken strongly predicting non-visual opsin expression by MCs. In fact, this was later confirmed in primary cultures of MCs, prepared at E8 and kept for 2 weeks, showing co-expression of GS/vimentin and Opn3/Opn5 (Figures 3, 4).

The presence of Opn3 in extra ocular tissue has been related to the light-driven regulation of different functions (Koyanagi et al., 2013) such as wound healing responses (Castellano-Pellicena et al., 2018) and pigmentation of melanocytes (Regazzetti et al., 2018). Although the precise role of Opn3 in the developing and mature retina of birds is still unknown, it is noteworthy that levels of Opn3 protein and its intracellular localization are highly regulated by light in MCs (Figure 4 and Supplementary Figure 3), neurons (data not shown), and melanocytes (Ozdeslik et al., 2019). Particularly, Opn3 protein levels in MCs were significantly induced by light exposure (1 h BL or 1 h post-BL) with a marked modification in protein pattern location, in which the immunolabeling associated to Opn3 substantially decreased in the cellular nuclei in the light condition and it was recovered 1 h later in the dark after BL exposure (Figure 4). In addition, it is important to remark that this mechanism of light induction and protein intracellular re-localization of Opn3 protein is dependent on *de novo* synthesis of protein as clearly observed after treatment with CHD (Supplementary Figure 3).

Opn5 mRNA was found to be expressed in mouse testis, brain, and eye, as well as in human retina and brain (Tarttelin et al., 2003). Whereas in the rat, Opn5 mRNA and protein are clearly expressed in the retina, specifically in INL and GCL cells and in IPL processes (Nieto et al., 2011); moreover, within the human and mice retina Opn5 is particularly seen in a subset of non-rod/non-cone retinal neurons and in the epidermal and muscle cells of the outer ears (Kojima et al., 2011). In the chicken, it was shown to be expressed in the pineal gland and in neurons of the INL and GCL of the retina of post-hatching chicks at PN14 (Yamashita et al., 2010), reflecting a similar retinal distribution in mammalian and non-mammalian retinas. Here we demonstrated that Opn5 mRNA and protein appeared very early in the developing retina at around E7–10, particularly in cells of the forming GCL and cells extending throughout the immature inner retina, resembling glial morphology (Figure 1). Moreover, primary cultures of MCs clearly exhibited a positive immunoreactivity for Opn5-like protein, further supporting the idea that this opsin is also expressed in retinal cells and MC cultures at early developmental stages (Figure 3). In fact, this opsin plays a key role during eye development in mice mediating light-dependent vascularization by a dopamine pathway (Nguyen et al., 2019). In the mature retina at PN10, Opn5-like protein is visualized in the GCL, INL, and processes concentrated at the IPL and OPL. These results allow us to hypothesize that Opn5 may play a key role in sensing UV light in the inner retina. Indeed, this opsin constitutes a functional UV-sensitive Gi-coupled bistable photopigment with maximal efficiency ∼420 nm, capable of conferring light-induced Ca^2+^ responses, cyclic AMP decrease, and MAPK phosphorylation after heterologous expression in HEK-293 cells (Sugiyama et al., 2014). In birds, apart from its potential role in the retina, Opn5 is involved in the photoreception required for seasonal reproduction, presumably acting as a deep brain photoreceptor molecule (Nakane et al., 2010; Yamashita et al., 2010). In mammals, Opn5 has been reported to be directly and specifically involved in the photic synchronization of the retinal clock (Buhr et al., 2015) and more recently to be associated with photoentrainment and phase shifting to UV light (Ota et al., 2018). However, in triple knockout mice lacking essential components of phototransduction signaling for rods, cones, and ipRGCs, minimal responses were observed following UV light stimulation, suggesting a very limited role for Opn5 in driving excitatory photic responses within the mouse retina (Hughes et al., 2016).

In order to have an active opsin photopigment, the PRC requires a vitamin A derivative available to work as chromophore since it is known that for most photopigments characterized such chromophores are retinoids that can be recycled in the retina itself or in the adjacent retinal pigment epithelium (Diaz et al., 2016, 2017; Morera et al., 2016). In this connection, the putative photoisomerase RGR found to be expressed in the inner chicken retina (Bailey and Cassone, 2004) and particularly in ipRGCs early in development is likely to be used by the different opsins (Opn3, 4, and 5) for the purpose of modulating retinaldehyde levels in light and thus keeping the balance of inner retinal retinoid pools (Diaz et al., 2016). Initially identified in bovine retinal pigment epithelium (Jiang et al., 1993), RGR was subsequently found within the retina in RGCs and/or MCs (Pandey et al., 1994; Hao and Fong, 1996, 1999; Chen et al., 2001a, b; Maeda et al., 2003; Bailey and Cassone, 2004; Wenzel et al., 2005; Diaz et al., 2017). It thus appears more likely that RGR enhances the classical and ipRGC visual cycles rather than forming part of an alternative photic visual cycle (Wenzel et al., 2005; Radu et al., 2008; Diaz et al., 2017).

### Retinal Müller Glial Cells as Light-Sensors: Ca^2+^ Responses Triggered by Light Stimulation

Here we show for the first time that primary cultures of embryonic MCs expressing typical markers for glia also display the expression of Opn3- and Opn5-like proteins (Figure 3). Similar results were observed in retinal sections having double immunolabeling for Opn3 and the glial marker GS (Figure 2). Additionally Opn3 levels and cellular localization are modified by BL stimulation by a mechanism dependent on protein synthesis (Figure 4 and Supplementary Figure 3). The question arising from these observations is whether these opsin-expressing glial cells respond to light. One known characteristic of vertebrate photosensitivity to PRCs, ipRGCs, and Opn4x-expressing HCs is the change in intracellular Ca^2+^ levels after light exposure, differentially causing cell hyperpolarization or depolarization, respectively, in avian and mammalian retinal cells (Qiu et al., 2005; Sekaran et al., 2005; Contin et al., 2010; Nieto et al., 2011; Meister and Tessier-Lavigne, 2013; Morera et al., 2016; Diaz et al., 2017). MCs primary cultures showed distinct and specific increases in somatic Ca^+2^ responses to a brief light stimulus for at least 20 s at the blue range wavelength (∼480 nm) (Figure 5) compared with basal values at dark conditions or cultures subject to shorter light exposure times and different wavelengths (red, white) (data not shown). Furthermore, three different subpopulations of cells were observed in the MCs primary cultures in terms of responses to BL: 41% of the population showed no response, whereas the 59% remaining displayed detectable BL responses; most of them responding with a >20% average increase in fluorescence. This latter segment of the population exhibited lasting responses to brief light pulses of at least 20 s, persisting for at least 100–300 s. In agreement with this, the photic responses involving Ca^+2^ mobilization were lost when cells were pretreated with hydroxylamine a retinaldehyde bleacher as previously shown in intrinsically photosensitive HCs strongly indicating that this phenomenon requires an active chromophore (Morera et al., 2016). Even though most cells expressed Opn3, only ∼60% of them responded to light with Ca^+ 2^ increase, the remaining percentage may not have functional opsins in their membrane; need longer time of exposure, higher BL intensities, or might be activating a different signaling pathway, i.e., related to cyclic nucleotides as it has been previously described by Koyanagi et al. (2013). Indeed, in *ex vivo* conditions there is only a percentage of MCs that show Ca^+ 2^ increase in response to the neuronal activation observed after light or electric stimulation (Newman, 2005). In line with our results the human MCs line MIO-M1 was shown to express a number of different opsins and to respond to repetitive stimulation with 480 nm light (Hollborn et al., 2011). In isolated rat retina, Ca^+ 2^ increases in glial cells were evoked by light-induced neuronal activity, suggesting a neuron-to-glia signaling in the retina mediated by ATP release (Newman, 2005). In MCs of the adult guinea pig retina, light stimulation evoked two differential Ca^+2^ responses (Rillich et al., 2009) probably an indication that retinal light stimulation causes glial activation by alterations in both the membrane potential and ATP-mediated mechanisms. However, our results clearly show direct Ca^+2^ responses to BL stimulation by MCs in primary cultures free of other types of retinal cells. Since Opn3 responds specifically to BL in a wavelength range peaking at 480 nm whereas Opn5 has been reported to respond to UV stimulation at 380 nm in the dark, we assumed that Opn5 would not be selectively activated by the experimental conditions applied (BL in dark adapted cells) (Kojima et al., 2011; Yamashita et al., 2014). Nevertheless, we cannot discard that the edges of the action spectra for lights centered at 480 nm could stimulate Opn5 in the violet/UV range as reported. Further experiments will be required to fully understand the role of retinal glial cells under physiological conditions and photic stimulation in the context of the entire retina. In this connection, Ca^+ 2^ increase in MCs has been linked to ATP release (Newman, 2003), extracellular H^+^ flux (Tchernookova et al., 2018), and potentially D-serine release (Metea and Newman, 2006), to regulate retinal neurotransmission. In addition, MCs may play a role in non-visual processes regulated by light such as the setting of the biological clock as well as in optical and visual functions acting as living optic fibers or enhancers of human vision acuity (MacDonald et al., 2017). Along these lines it has recently been demonstrated that a cell-autonomous clock present in SCN astrocytes may drive circadian behavior in mammals (Brancaccio et al., 2019).

Overall, we show that the two studied non-visual opsins are present in the inner retina of chicks from very early developmental stages to PN before there is any sign of vision. Their expression was found to be present in inner retinal cells including MCs kept in culture for several days. It is therefore likely that these opsins contribute with Opn4m and x to the detection of light and thus to regulation of the diverse activities required for the adequate development of the eye and functioning of the avian retina. Moreover, we demonstrated that MCs were affected by BL stimulation in terms of Opn3 protein and its intracellular localization as previously reported for the immunoreactivity of Opn4 and Opn5 in the retina of rats exposed to continuous light of low intensity (Benedetto et al., 2017). In addition, similar results were found in primary cultures of Opn3 (+) retinal neurons of chick embryos exposed to BL and displaying marked changes in protein levels and intracellular localization (data not shown). More important, MCs expressing non-visual opsins appear to be able to sense BL in the inner retina by increasing intracellular levels of Ca^2+^, novel events that presumably affect cell to cell interaction and glia to neuron communication, while increasing levels of Opn3 in the whole cell, thus preparing the inner retina and its circuits for the light exposure times.

## Data Availability

The datasets generated for this study are available on request to the corresponding author.

## Ethics Statement

All experiments were performed in accordance with the Use of Animals in Ophthalmic and Vision Research of ARVO, approved by the local animal care committee (School of Chemistry, National University of Córdoba; Exp. 15-99-39796) and CICUAL (Institutional Committee for the Care and Use of Experimental Animals).

## Author Contributions

MR, NM, and MG designed the research, and analyzed and discussed the data. MR and NM performed the research. MG contributed to the new reagents, analytic tools and wrote the manuscript.

## Conflict of Interest Statement

The authors declare that the research was conducted in the absence of any commercial or financial relationships that could be construed as a potential conflict of interest.

## Footnotes

1 https://www.proteinatlas.org/ENSG00000054277-OPN3/cell#human
