# Supplementary figures and images for: Expression of Non-visual Opsins Opn3 and Opn5 in the Developing Inner Retinal Cells of Birds. Light-Responses in Müller Glial Cells
Source: Front Cell Neurosci. 2019 Aug 16;13:376. doi: 10.3389/fncel.2019.00376 (PMC6706981; doi:10.3389/fncel.2019.00376)

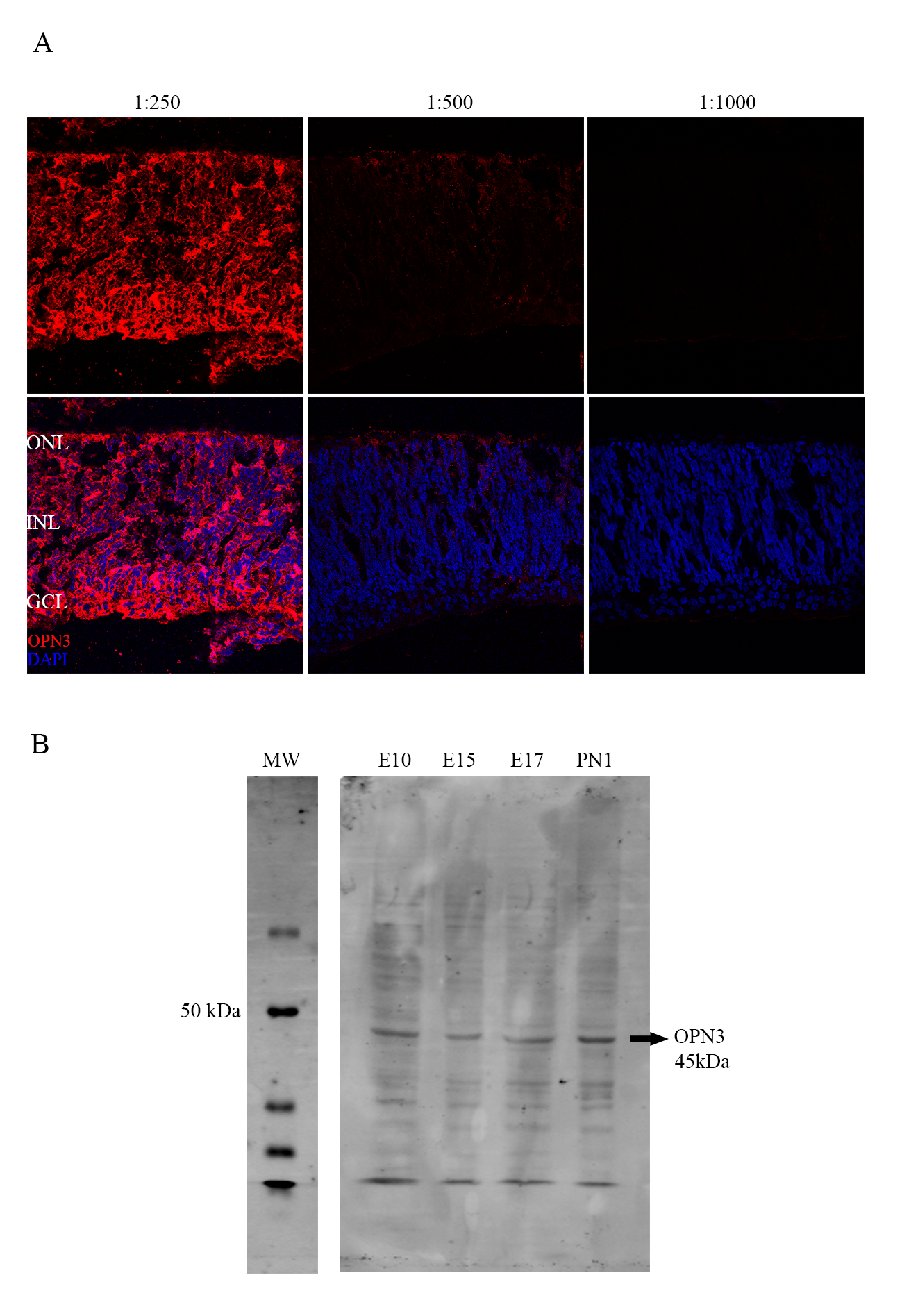

Supplement: Supplementary file 1 [file Image_1.TIF]

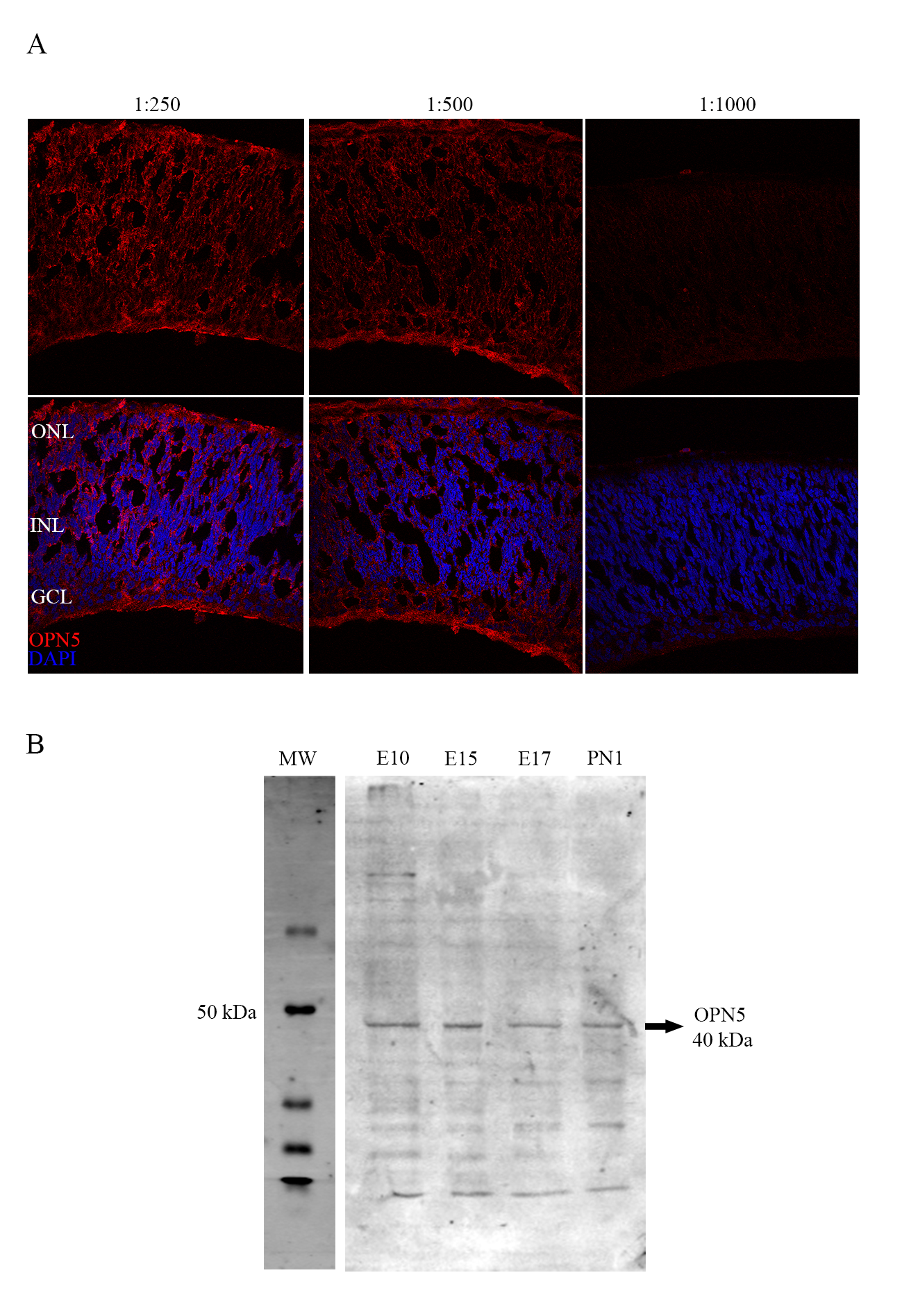

Supplement: Supplementary file 2 [file Image_2.TIF]

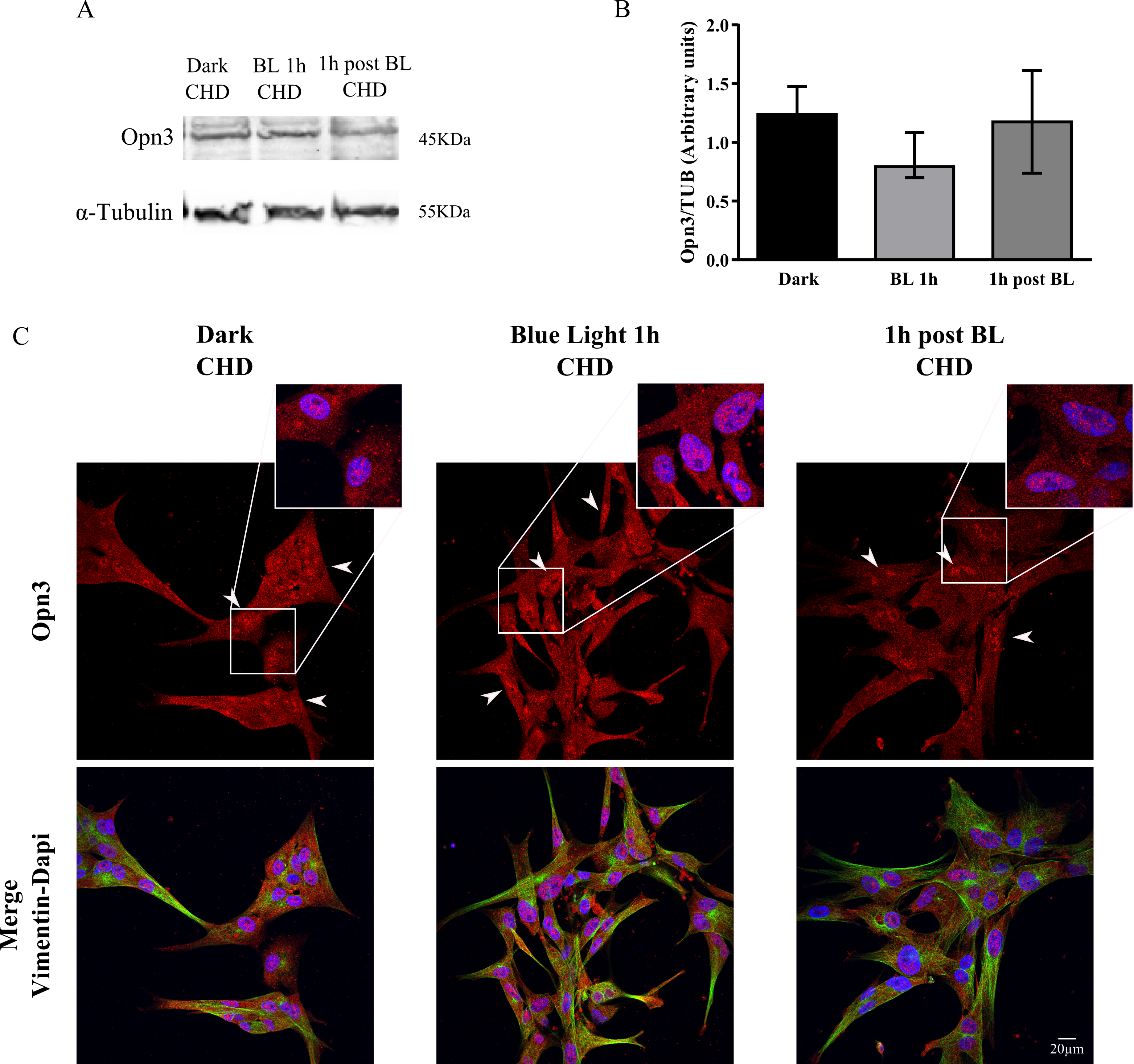

Supplement: Supplementary file 3 [file Image_3.TIF]

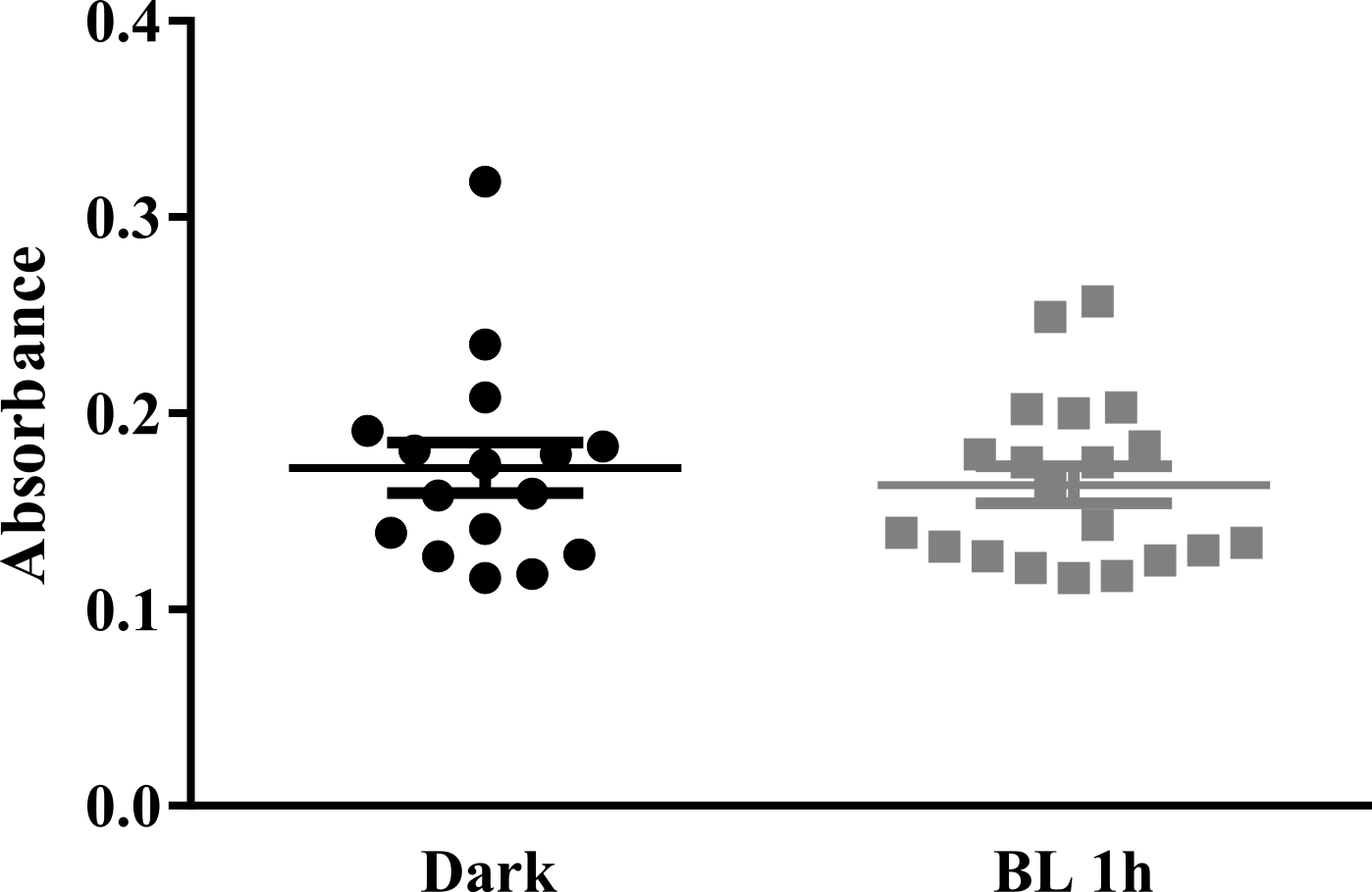

Supplement: Supplementary file 4 [file Image_4.TIF]

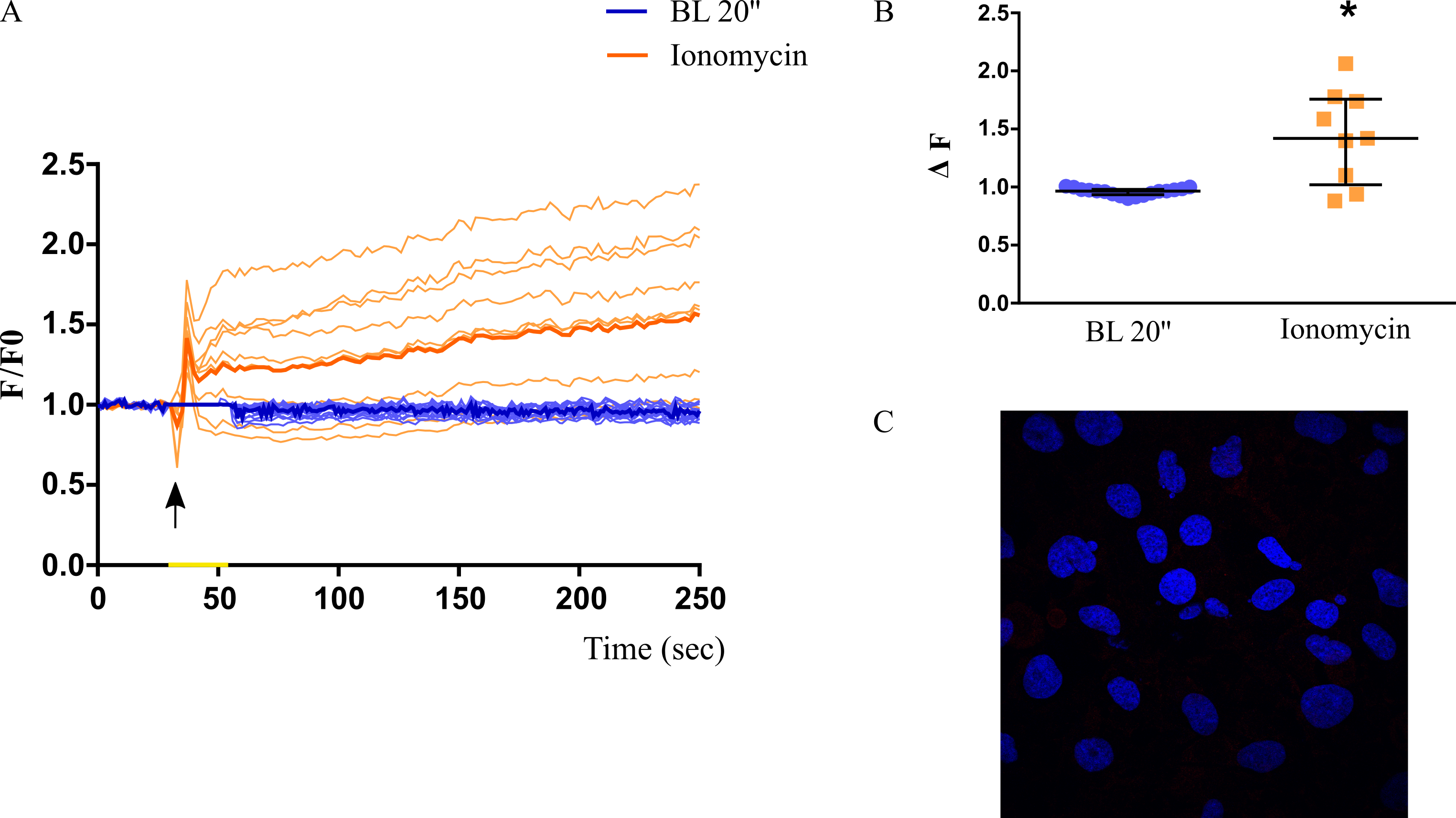

Supplement: Supplementary file 5 [file Image_5.TIF]
