# Supplementary material for: Expression of Non-visual Opsins Opn3 and Opn5 in the Developing Inner Retinal Cells of Birds. Light-Responses in Müller Glial Cells
Source: Front Cell Neurosci. 2019 Aug 16;13:376. doi: 10.3389/fncel.2019.00376 (PMC6706981; doi:10.3389/fncel.2019.00376)
Supplement: Supplementary file 6 [file Table_1.DOCX]

**Supplementary Figure 1. Specificity Analysis for the primary antibody against Opn3 protein.**

(A) Dilution curve for Opn3-like protein (red) immunohistochemistry in retinal sections from chicken embryos at E10. Dilutions tested with the specific primary antibody were 1:250; 1:500 and 1:1000. Nuclear staining with DAPI (blue). ONL: outer nuclear layer, INL: inner nuclear layer, GCL: ganglion cell layer

(B) Western blot with the complete immunoblot for Opn3 protein with the specific primary antibody that detected the corresponding band at ∼43-45 kDa for total retina homogenates from E10 to PN1.

**Supplementary Figure 2. Specificity Analysis for the primary Opn5 protein antibody.**

(A) Dilution curve for Opn5-like protein (red) immunohistochemistry in retinal sections from chicken embryos at day 10. Dilutions tested with the specific primary antibody were 1:250; 1:500 and 1:1000. Nuclear staining with DAPI (blue). ONL: outer nuclear layer, INL: inner nuclear layer, GCL: ganglion cell layer

(B) Western blot with the complete immunoblot for Opn5 protein with the specific primary antibody that detected the corresponding band at ∼40 kDa for total retina homogenates from E10 to PN1.

**Supplementary Figure 3.** **Changes in Opn3 expression by blue light treatment in Müller glial cells depend on protein synthesis**

Primary cultures of Müller glial cells were pretreated with cycloheximide (CHD; 50μg/ml) for 1 h before 1 h of blue light (BL) stimulation, or at the beginning of the blue light stimulus for the 1 h post BL group. (A) Immunoblots of Opn3 protein in the three experimental conditions (Dark, BL 1h and 1 h post BL). (B) The graph shows the median value with range for Opn3 expression. No increase in Opn3 levels was observed after BL stimulation or 1h post BL when cells were pretreated with CHD (K-W test, H_(2)_= 0.27 non-significant (N.S.) n=3/group). (C) Opn3-like protein immunoreactivity (red) in Müller glial cell cultures also stained for the glial marker Vimentin-like protein (green) and DAPI (blue). No intracellular changes in localization of Opn3 immunofluorescence were observed in the different conditions examined after CHD treatment retaining nuclear accumulation (marked with arrowheads) of Opn3 like-protein in all CHD pre-treated groups. Insets further magnified the nuclear immunostaining of Opn3-like protein.

**Supplementary Figure 4. Blue light stimulation does not promote cell death inprimary cultures of** **Müller glial cells**

Graphical representation (media ± SEM) of MTT reagent absorbance of Müller glial cell cultures in darkness or blue light (BL) for 1 h. BL stimulation for 1h to Müller cells in culture does not promote cell death, when measured 24 h after the stimulus; t_(34, 0.05)_=0.55 N.S. (n=16-20/condition).

**Supplementary Figure 5. Intracellular Ca^2+^ levels response to blue light and ionomycin treatment in HEK-293 cells**

(A) Graphical representation showing the F/Fo ratio for different individual Ca^2+^ responses in HEK-293 cells. Blue lines: Ca^2+^ responses when cells were exposed to a single brief blue light (BL) pulse of 68 µW/cm^2^ for 20 sec (indicated with yellow mark in the X axis); the dark blue line represents the average response to BL. Orange lines: Ca^2+^ responses when cells were supplemented with ionomycin (2 µM) (no BL exposure); the dark orange line represents the average response to ionomycin stimulation. (B) Graphical representation of relative fluorescent Ca^2+^ levels (ΔF) in HEK-293 cells when exposed to a blue light pulse (BL20’’) or ionomycin. The graph shows the median value with range; M-W U_(17,9,0.05)_= 30 *p<0.05; (26 cells recorded in 4 independent experiments). (C) Opn3-like protein immunoreactivity (red) in HEK-293 cell cultures with nuclear staining by DAPI (blue). No Opn3 immunoreactivity was visualized in these cells.
