# Supplementary material for: Expression of Non-visual Opsins Opn3 and Opn5 in the Developing Inner Retinal Cells of Birds. Light-Responses in Müller Glial Cells
Source: Front Cell Neurosci. 2019 Aug 16;13:376. doi: 10.3389/fncel.2019.00376 (PMC6706981; doi:10.3389/fncel.2019.00376)
Supplement: Supplementary file 7 [file Table_2.DOCX]

**Supplementary Table 1. List of antibodies.**

| **Primary antibody** | **ICC /IHC** | **WB** | **CAT#** |
| --- | --- | --- | --- |
| Opn3 | 1:500 | 1:500 | **(NovusCat# NB110-74721, RRID:AB_2158340)** |
| Opn5 | 1:500 | - | **(NovusCat# NBP1-00943, RRID:AB_1503763)** |
| α-Tubulin | 1:1000 | 1:2000 | **(Sigma-Aldrich Cat# T9026, RRID:AB_477593)** |
| Glutamine synthase (GS) | 1:500 | 1:1000 | **(Millipore Cat# MAB302, RRID:AB_2110656)** |
| Vimentin | 1:1000 | 1:1000 | **(Sigma-Aldrich Cat# V5255, RRID:AB_477625)** |
| GFAP | - | 1:1000 | **(Sigma-Aldrich Cat# G9269, RRID:AB_477035)** |

Summary of antibodies used indicating the dilution for each molecular technique.

ICC: immunocytochemistry; IHC: immunohistochemistry; WB: western blot; CAT#: catalogue number
